# Supplementary material for: Emergent Chaos‐Like Dynamics of Spin–Orbit‐Torque‐Driven Magnetic Transitions
Source: Small. 2026 May 14;22(37):e73778. doi: 10.1002/smll.73778 (PMC13325707; doi:10.1002/smll.73778)
Supplement: Supplementary file 1 — Supporting File 1: smll73778‐sup‐0001‐SuppMat.pdf. [file SMLL-22-e73778-s001.pdf]

Supplementary Information to Emergent Chaos-Like Dynamics of Spin–Orbit-Torque-Driven Magnetic Transitions

L.-M. Kern*†,[1] K. Litzius†,[2] V. Deinhart,[3,4] M. Schneider,[1] C. Klose,[1] K. Gerlinger,[1] R. Battistelli,[3] D. Metternich,[3] D. Engel,[1] C. M. Günther,[5] M.-J. Huang,[6] K. Höflich,[4,3] F. Büttner,[2,3] S. Eisebitt,[1,7] and B. Pfau[1]

[1]*Max Born Institute for Nonlinear Optics and Short Pulse Spectroscopy, 12489 Berlin, Germany**

[2]*Experimental Physics V, Center for Electronic Correlations and Magnetism, University of Augsburg, 86159 Augsburg, Germany*†

[3]*Helmholtz-Zentrum Berlin für Materialien und Energie, 14109 Berlin, Germany*

[4]*Ferdinand-Braun-Institut (FBH), 12489 Berlin, Germany*

[5]*Technische Universität Berlin, Zentraleinrichtung Elektronenmikroskopie, 10623 Berlin, Germany*

[6]*Deutsches Elektronen-Synchrotron, 22607 Hamburg, Germany*

[7]*Technische Universität Berlin, Institut für Optik und Atomare Physik, 10623 Berlin, Germany*

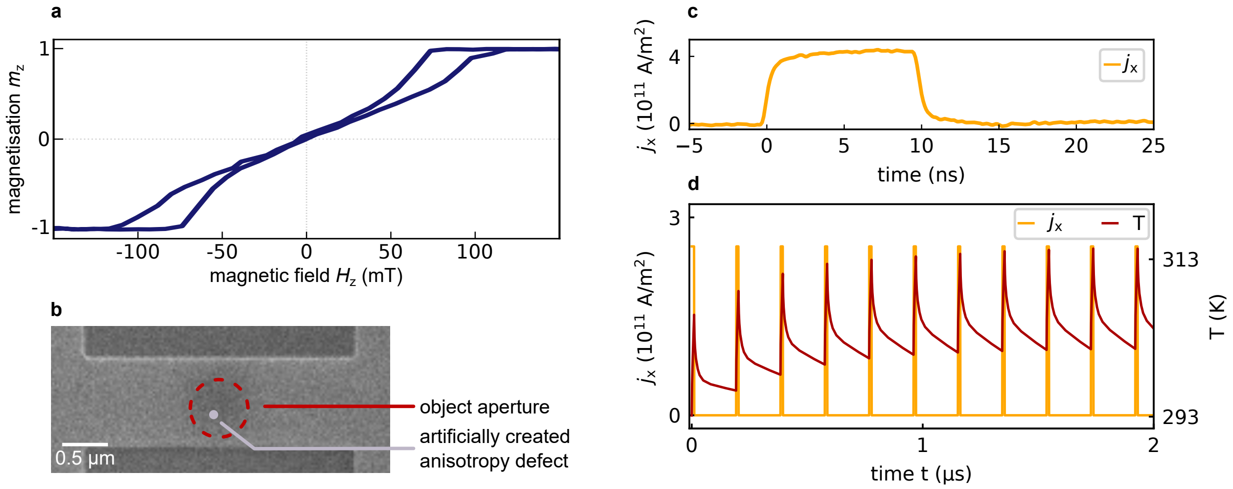

Supplementary Fig. 1. **Experimental Details.** **a** Hysteresis curve of [Pt/CoFeB/MgO]-multilayer, recorded using magneto-optical Kerr microscopy. **b** Scanning electron microscopy image of the magnetic racetrack with an object aperture of 600 nm diameter (red) and an ion-irradiated anisotropy-engineered region of 100 nm diameter (purple). **c** Rectangular current pump pulses of 10 ns duration are injected into the device, synchronized to the bunch clock at a repetition rate of 5.2 MHz. The delay between current pump and x-ray probe beam is variable. **d** Simulations of the current-induced temperature evolution estimate a peak temperature of 22 K above room temperature and a static heating of 9 K of the magnetic film.

I. EXPERIMENTAL DETAILS

Using magneto-optical Kerr microscopy, we measured the magnetic hysteresis curve of our [Pt/CoFeB/MgO] multilayer (Supplementary Fig. 1a). Supplementary Fig. 1b shows a scanning electron micrograph of the magnetic stripline, the object aperture, and the position of the ion-irradiated dot. For the time-resolved experiment, rectangular unipolar 10 ns current pulses (Supplementary Fig. 1c) were injected into the stripline, synchronized to the x-ray probe at a 5.2 MHz repetition rate, with a variable pump–probe delay. Simulations of current-induced heating indicate a peak temperature rise of 22 K above room temperature, with a static increase of 9 K in the magnetic film (see Methods for simulation details).

* Corresponding author: kern@mbi-berlin.de

† These authors contributed equally to this work.

II. TIME-RESOLVED IMAGING

In this Supporting Information, we present additional time-resolved measurements that complement the results shown in the main text. Specifically, we illustrate the deformation, stripe-out, and fluctuation regimes by comparing experimental and simulated image frames side by side. For each panel, the applied magnetic field and current density used in the experiment (top row) and simulation (bottom row) are indicated, and the color code corresponds to the dynamics introduced in Fig. 2 (see main text and Methods for details).

In the deformation regime (Supplementary Fig. 2a), spin-orbit torque (SOT) from a low current density leaves the skyrmion largely unchanged during and after the pulse, consistent with the subtle deformation seen in the simulations. The magnetization contrast is preserved, providing no evidence for transient chaotic behavior at this current density.

In the stripe-out regime (Supplementary Fig. 2b), the magnetization extends outward, forming a tail of reduced contrast outside the dot. By the end of the pulse, the contrast within the dot itself is also reduced, indicating more pronounced dynamics.

In the fluctuation regime (Supplementary Fig. 2c), transient chaotic dynamics are observed, but they remain confined to the area of the dot, highlighting the localized and stochastic nature of these magnetization fluctuations.

In addition, Supplementary Fig. 3 presents simulations of the pump–probe scheme including thermal spin fluctuations alongside the experimental observations. Supplementary Fig. 3a shows the experimental fluctuation regime from Fig. 4. Supplementary Fig. 3b shows seven consecutive SOT pulses simulated with thermal noise, with the first pulse preparing the initial state. Thermally induced fluctuations subtly modify the initial conditions for each pulse, analogous to small variations in current density (see Fig. 4b). Supplementary Fig. 3c demonstrates that these varying trajectories lead to an overall washed-out magnetization contrast, illustrated by overlaying images from six consecutive pulses.

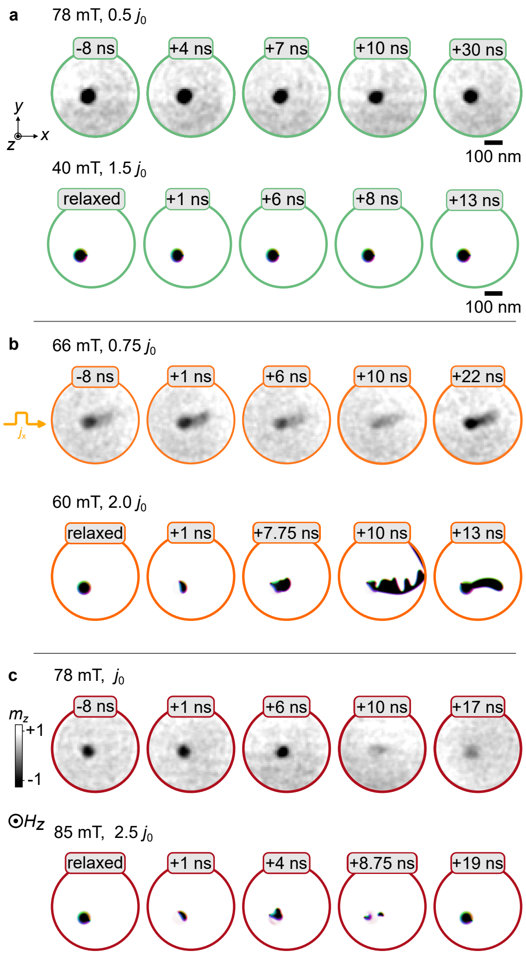

Supplementary Fig. 2. **Time-resolved imaging of deformation, stripe-out and fluctuation dynamics.** The applied field and the current density used in the experiment (top row in each panel) and simulation (bottom row) are indicated. The colour code corresponds to the dynamics introduced in Fig. 2. The FOV in the experiment is fixed to a circular region (diameter 600 nm) (see main text and Methods for details). **a** Deformation. SOT from a low current density leaves the skyrmion almost unchanged during and after the current pulse, in line with the subtle deformation dynamics found in the simulations. Since the magnetization contrast is not reduced, there is no experimental evidence for transient chaotic behavior at this current density. Note that the experiment was performed at $j_x = 0.5j_0$, which is considerably below the single-pulse nucleation threshold j_0 (see Methods for details on j_0). The initial state was therefore prepared with a single current pulse with $j_x = j_0$. **b** Stripe-out. The magnetization stripes out and forms a tail of reduced contrast outside the dot. Towards the end of the pulse, also the contrast in the dot is clearly reduced. **c** Fluctuations. Full size of the images shown in Fig. 4. The transient chaotic dynamics are restricted to the area of the dot.

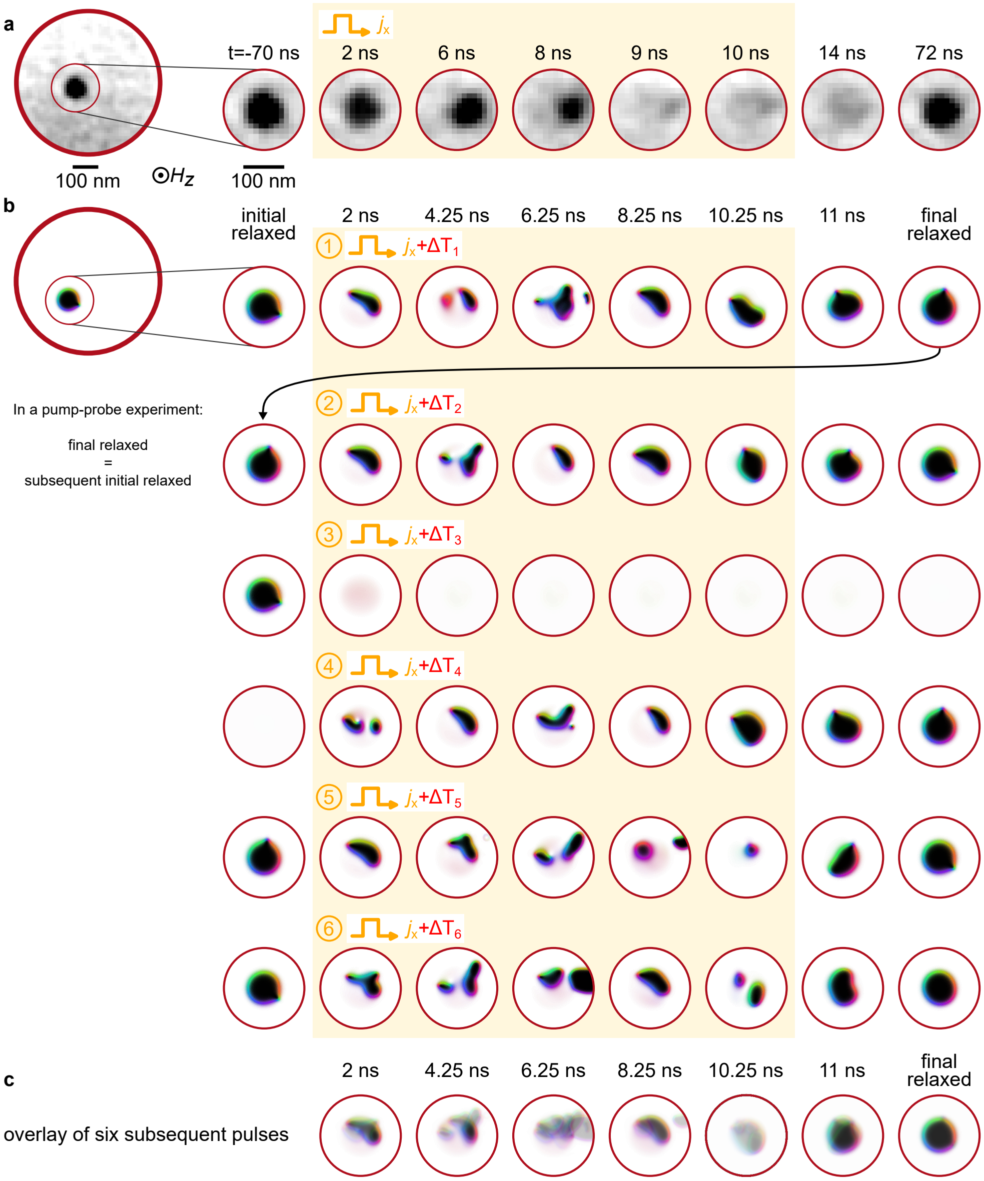

Supplementary Fig. 3. **Simulation of pump-probe scheme including thermal spin fluctuations with overlayed magnetization images.** **a** Experimental observation in the fluctuation regime showing reduced magnetization contrast near the end of the SOT drive and shortly after. **b** Seven consecutive SOT pulses with thermal noise; the first pulse (not shown) prepares the initial state. Thermally induced spin fluctuations subtly alter the initial conditions for each pulse, similar to the slight variation in current density (Fig. 4b). **c** The varying trajectories result in an overall washed-out magnetization contrast, demonstrated by the overlay of images from six pulses.
