# Supplementary figures and images for: Emergent Chaos‐Like Dynamics of Spin–Orbit‐Torque‐Driven Magnetic Transitions
Source: Small. 2026 May 14;22(37):e73778. doi: 10.1002/smll.73778 (PMC13325707; doi:10.1002/smll.73778)

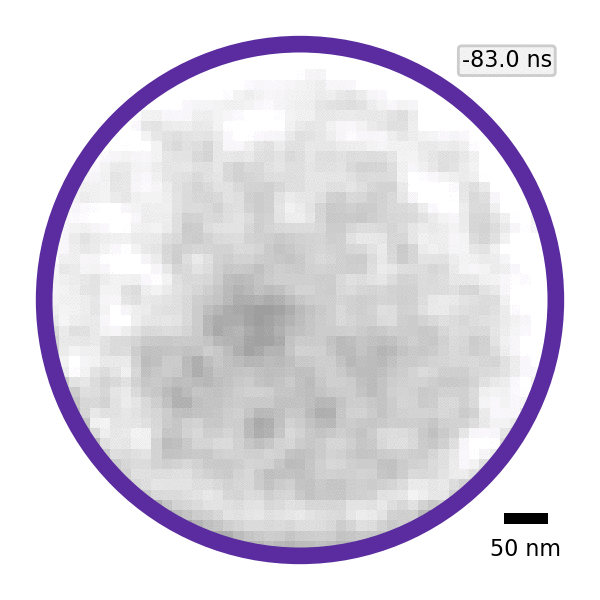

Supplement: Supplementary file 2 — Supporting File 2: smll73778‐sup‐0002‐MoviesS1.zip. [file SMLL-22-e73778-s002.zip › 3_exp_canting.gif]

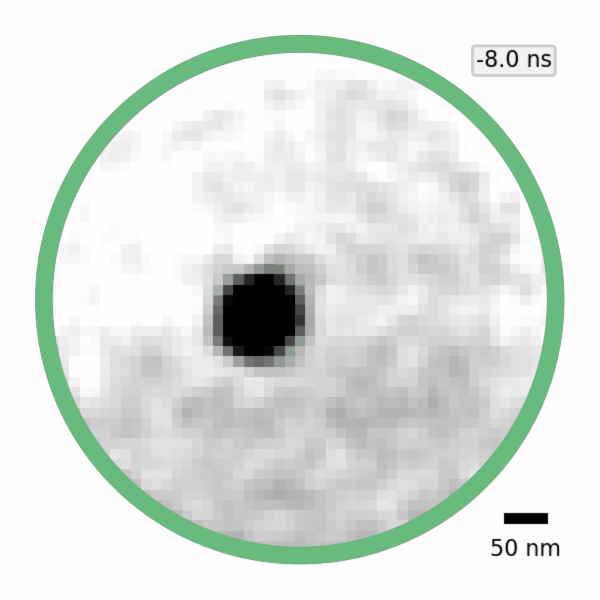

Supplement: Supplementary file 2 — Supporting File 2: smll73778‐sup‐0002‐MoviesS1.zip. [file SMLL-22-e73778-s002.zip › 3_exp_deformation.gif]

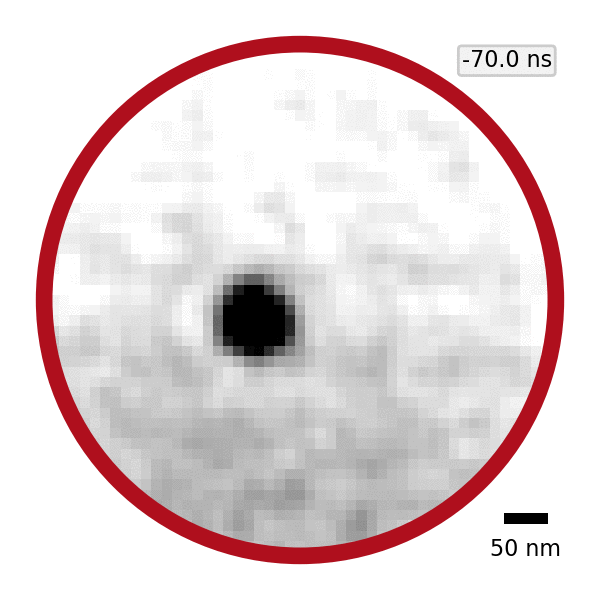

Supplement: Supplementary file 2 — Supporting File 2: smll73778‐sup‐0002‐MoviesS1.zip. [file SMLL-22-e73778-s002.zip › 3_exp_fluctuations.gif]

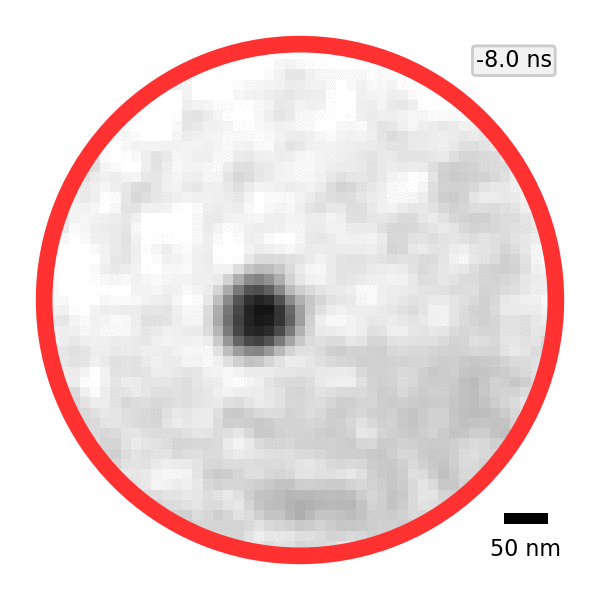

Supplement: Supplementary file 2 — Supporting File 2: smll73778‐sup‐0002‐MoviesS1.zip. [file SMLL-22-e73778-s002.zip › 3_exp_skyrmion_shedding.gif]

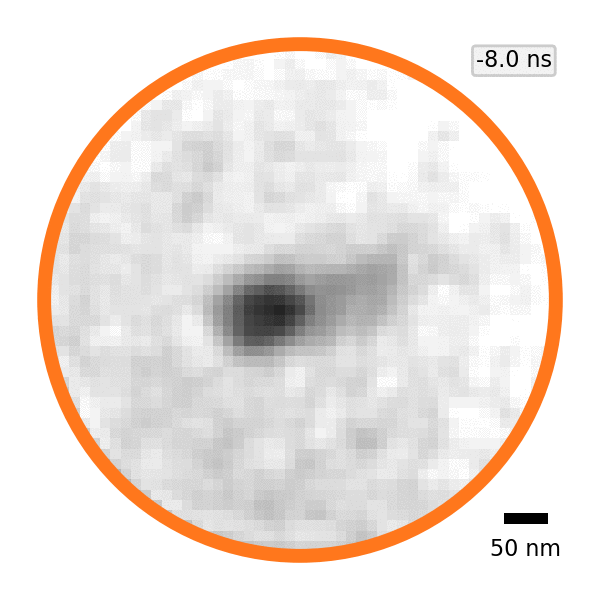

Supplement: Supplementary file 2 — Supporting File 2: smll73778‐sup‐0002‐MoviesS1.zip. [file SMLL-22-e73778-s002.zip › 3_exp_stripe-out.gif]

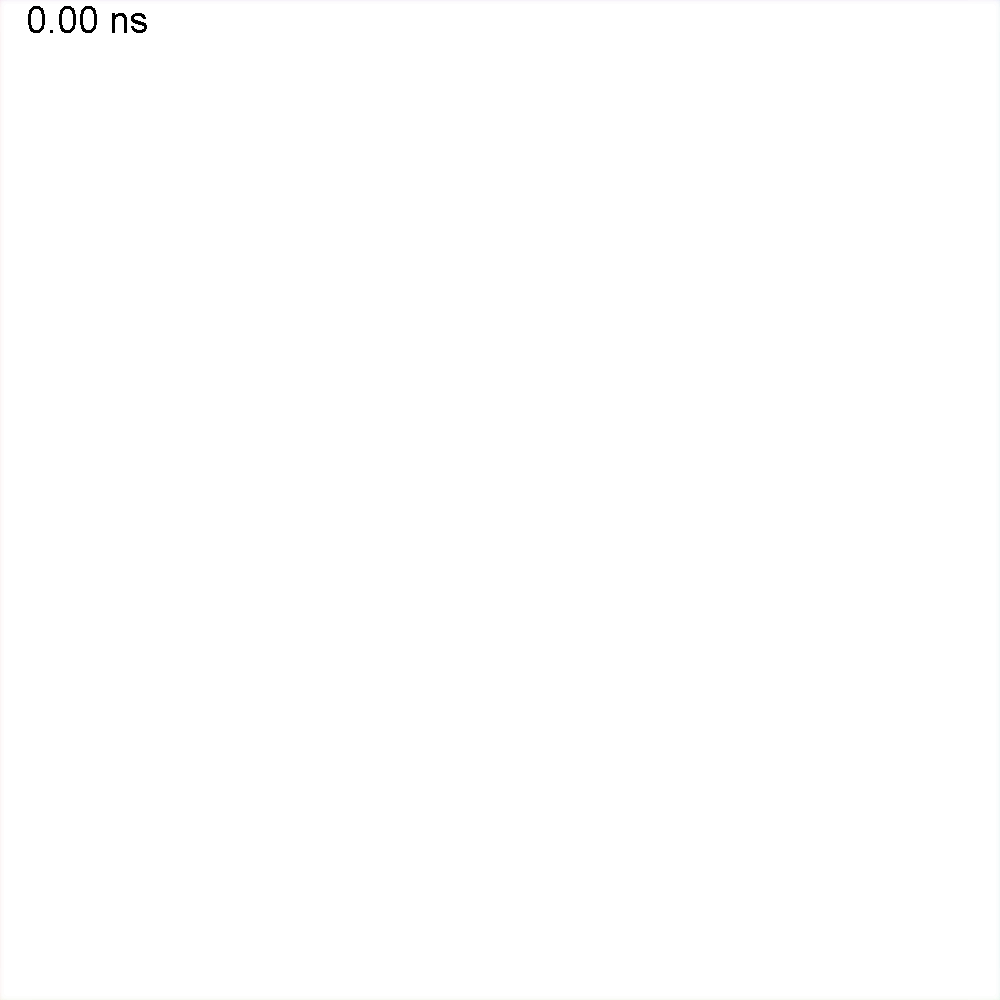

Supplement: Supplementary file 2 — Supporting File 2: smll73778‐sup‐0002‐MoviesS1.zip. [file SMLL-22-e73778-s002.zip › 3_sim_canting.gif]

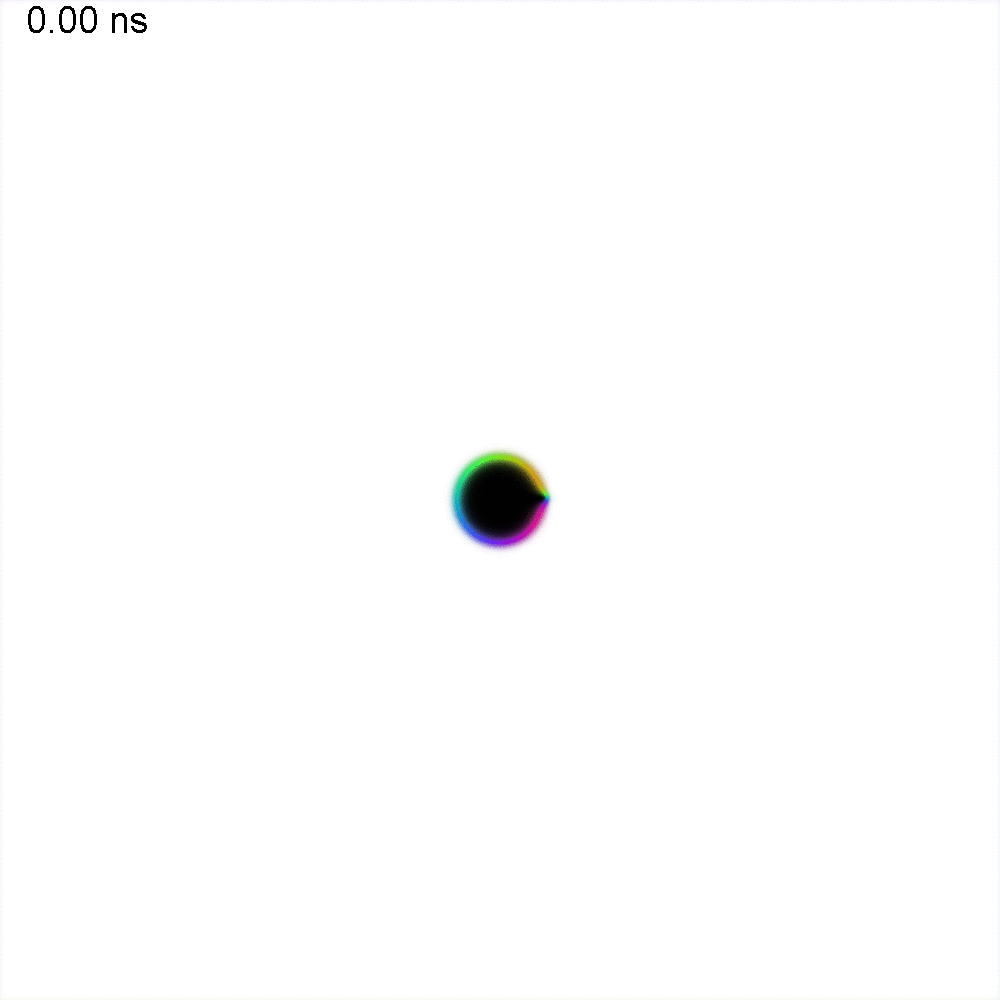

Supplement: Supplementary file 2 — Supporting File 2: smll73778‐sup‐0002‐MoviesS1.zip. [file SMLL-22-e73778-s002.zip › 3_sim_deformation.gif]

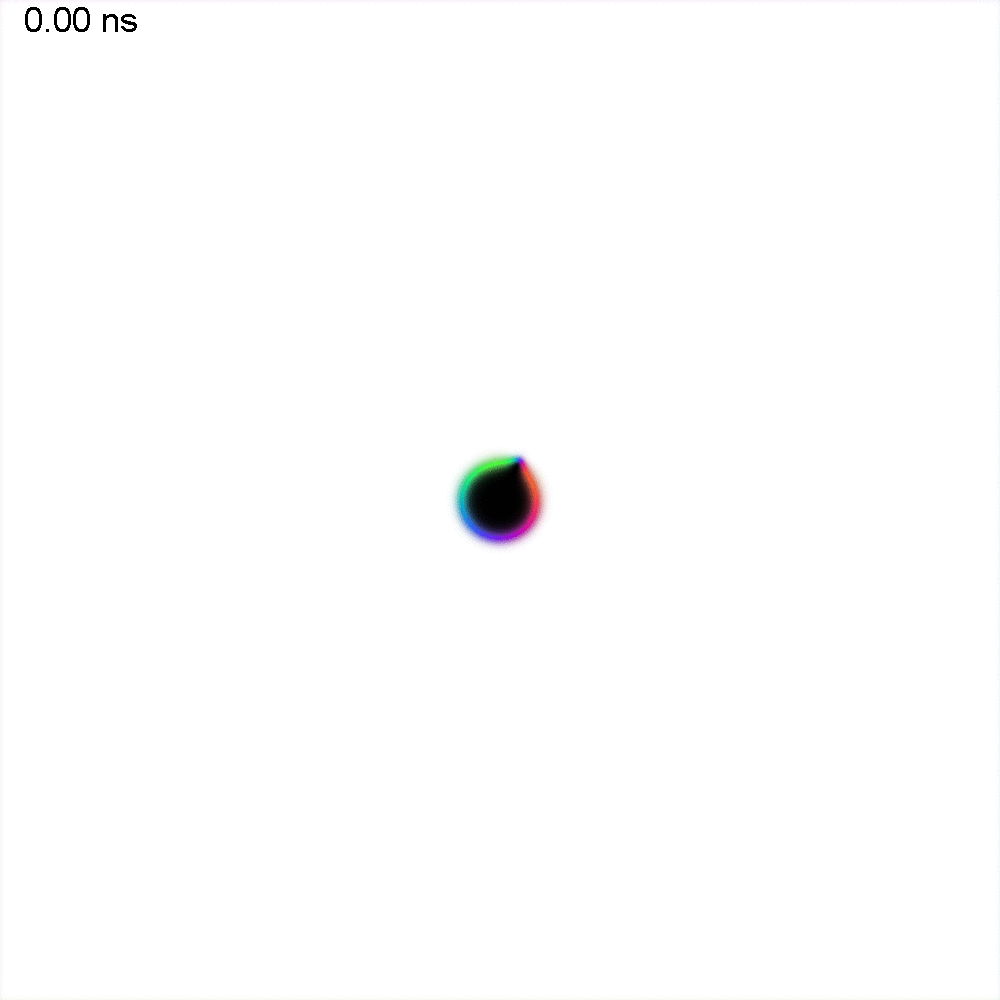

Supplement: Supplementary file 2 — Supporting File 2: smll73778‐sup‐0002‐MoviesS1.zip. [file SMLL-22-e73778-s002.zip › 3_sim_fluctuations.gif]

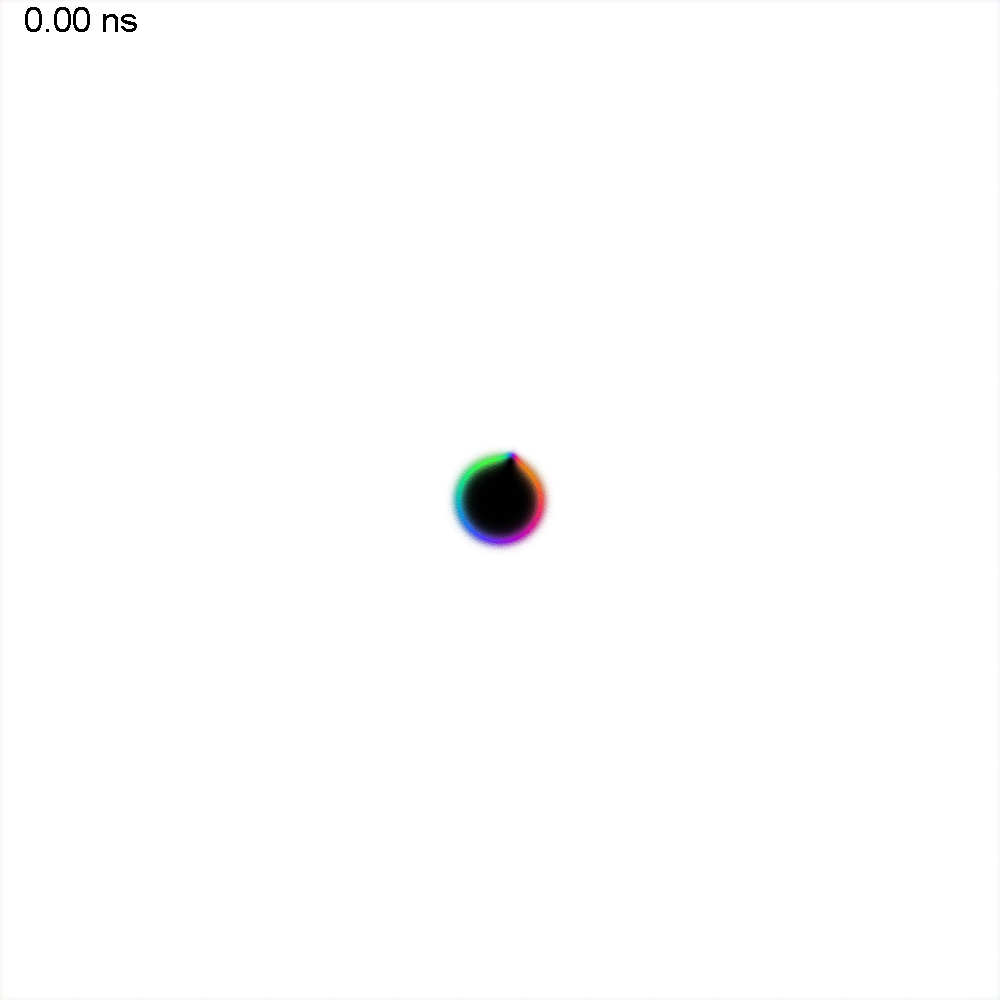

Supplement: Supplementary file 2 — Supporting File 2: smll73778‐sup‐0002‐MoviesS1.zip. [file SMLL-22-e73778-s002.zip › 3_sim_skyrmion_shedding.gif]

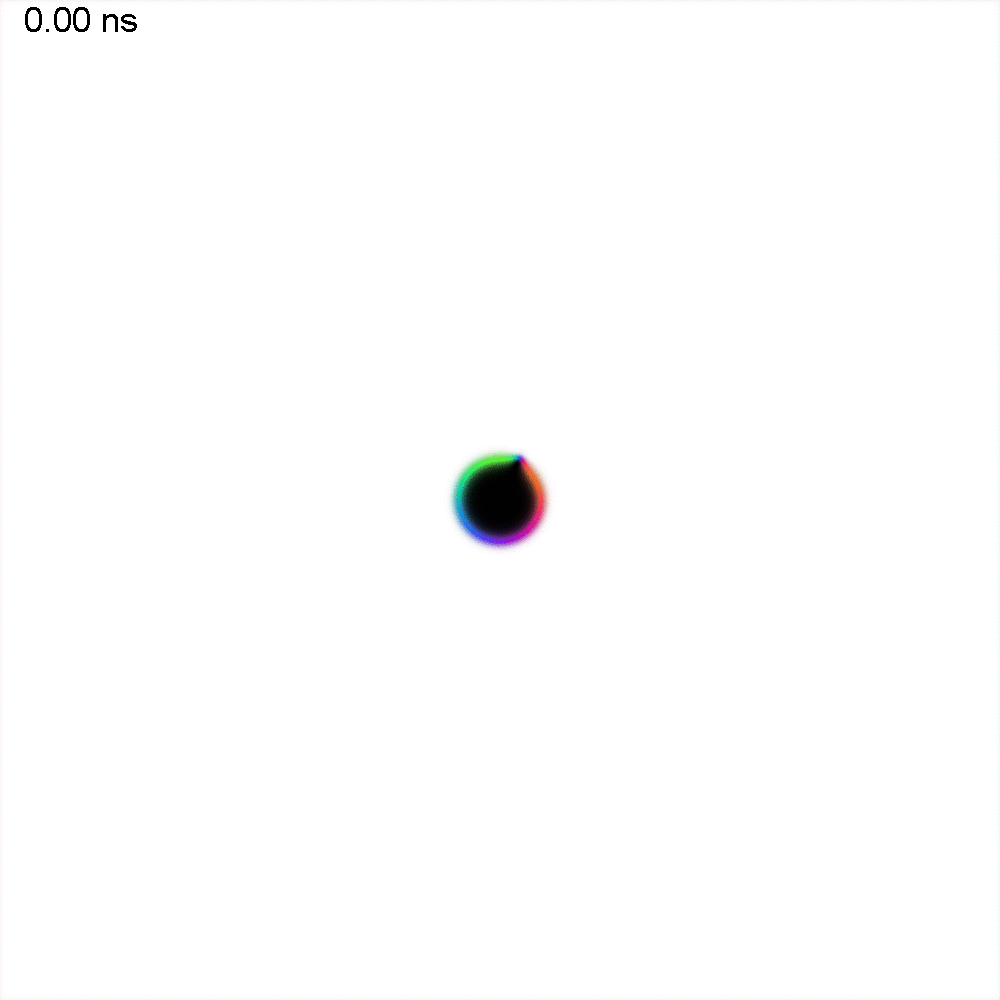

Supplement: Supplementary file 2 — Supporting File 2: smll73778‐sup‐0002‐MoviesS1.zip. [file SMLL-22-e73778-s002.zip › 3_sim_stripe-out.gif]
